# Correlation between diabetes mellitus and refracture risk in patients with osteoporotic fractures: a retrospective cohort study

**DOI:** 10.1007/s40520-024-02917-1

**Published:** 2025-03-13

**Authors:** Shao-han Guo, Jian Xu, Min-zhe Xu, Chong Li, Ya-qin Gong, Ke Lu

**Affiliations:** 1 Department of Orthopedics, Affiliated Kunshan Hospital of Jiangsu University, No. 566 East of Qianjin Road, Suzhou, Jiangsu 215300 China; 2 Department of Orthopedics, Gusu School, The First People’s Hospital of Kunshan, Nanjing Medical University, Suzhou, Jiangsu China; 3 Information Department, Affiliated Kunshan Hospital of Jiangsu University, Suzhou, Jiangsu China

**Keywords:** Diabetes, Osteoporotic fractures, Refracture risk, Retrospective cohort study

## Abstract

**Background:**

Diabetes and osteoporosis are frequent long-term conditions. There is little information on the relationship between diabetes and the risk of refracture in people who have osteoporotic fractures (OPFs), even though both conditions have been individually associated with increased fracture risk.

**Methods:**

We conducted a retrospective cohort study using the Osteoporotic Fracture Registry System of the Affiliated Kunshan Hospital of Jiangsu University. The study included 2,255 patients aged 50 years or older who were admitted with OPFs, comprising 107 with diabetes and 2,148 without. The risk of refracture within 1, 3, and 5 years was evaluated using Cox proportional hazard regression models based on whether or not a diabetes diagnosis was made during the admission assessment. Furthermore, the rates of refracture between individuals with and without diabetes were compared using Kaplan-Meier curves.

**Results:**

In patients with OPFs, diabetes was significantly positively correlated with refracture risk. For the follow-up periods of 1, 3, and 5 years, the hazard ratios (HRs) in the fully adjusted model were 2.83 (95% confidence interval [CI]: 1.09 to 7.39, *P*-value = 0.033), 2.65 (95% CI: 1.27 to 5.52, *P*-value = 0.009), and 2.72 (95% CI: 1.39 to 5.32, *P*-value = 0.004), respectively.

**Conclusions:**

The findings highlight the importance of monitoring bone health and implementing preventative interventions in individuals with diabetes, since they reveal that diabetic patients face a risk of refracture that is more than twice as high as that of non-diabetic individuals.

**Supplementary Information:**

The online version contains supplementary material available at 10.1007/s40520-024-02917-1.

## Introduction

Osteoporosis (OP) is a prevalent chronic skeletal disorder characterized by decreased bone mineral density (BMD) and deterioration of bone microarchitecture. These changes increase the possibility of fractures [1]. Refracture refers to any subsequent osteoporotic fracture that occurs following an initial fracture, regardless of whether it affects the same anatomical site or a different one [2]. It is called a secondary fracture at times. Osteoporotic fractures (OPFs) increase the risk, morbidity, and mortality of refractures and significantly burden the healthcare system financially and medically [3]. Among individuals with an incident fracture, the risk of a refracture increases by 30–40% within 3 years [4]. Post-fracture evaluation and pharmacological treatment are uncommon for individuals with fragility fractures [5, 6], even though bone-protective medicines can be utilized to lower refracture risk [7, 8].

Diabetes mellitus (DM) is a chronic metabolic condition characterized by elevated blood sugar levels resulting from inadequate insulin synthesis, insulin resistance, or both [9]. With an estimated 537 million adults affected globally in 2021 and a predicted increase to 783 million by 2045 [10], diabetes was the eighth greatest cause of death and disability combined in the world [11]. Diabetes also places a significant financial strain on healthcare systems [12, 13]. According to estimates from the International Diabetes Federation (IDF), global healthcare spending is expected to increase from $966 billion in 2021 to $1.054 trillion by 2045 [14]. Diabetes is recognized to be linked to several complications, such as nephropathy, neuropathy, retinopathy, and cardiovascular diseases [15, 16].

Several mechanisms may contribute to the increased risk of refracture in patients with diabetes. First, diabetes can impair bone quality due to the accumulation of advanced glycation end products (AGEs) in bone collagen, which reduces bone strength and increases fragility [17]. Second, hyperglycemia disrupts bone metabolism by inhibiting the maturation and functionality of osteoblast and osteoclast [18]. Third, diabetes-related microvascular complications may compromise bone blood supply and impede healing processes [19]. Additionally, diabetic neuropathy can increase fall risk by impairing balance and proprioception [20], while diabetic nephropathy may affect vitamin D metabolism and calcium homeostasis [21]. Collectively, these mechanisms highlight the substantial impact of diabetes on both initial fracture healing and the risk of subsequent refracture.

Diabetes-related fractures present considerable clinical challenges and can significantly reduce quality of life. To manage and avoid recurring fractures, it is critical to understand the link between diabetes and refractures. Nevertheless, evidence is scarce regarding the correlation between diabetes and the likelihood of refracture. A recent study investigated the correlation between diabetes and the probability of fractures in senior men in Germany [22]. Another pertinent study analyzed a population in Denmark [23]. Due to the limited research on osteoporotic fractures in the Chinese population, particularly with a follow-up duration of up to 5 years, our objective is to carry out a study on the correlation between diabetes and the risk of refracture in a representative group of hospitalized patients with OPFs.

## Materials and methods

### Study design and participants

**Fig. 1:**
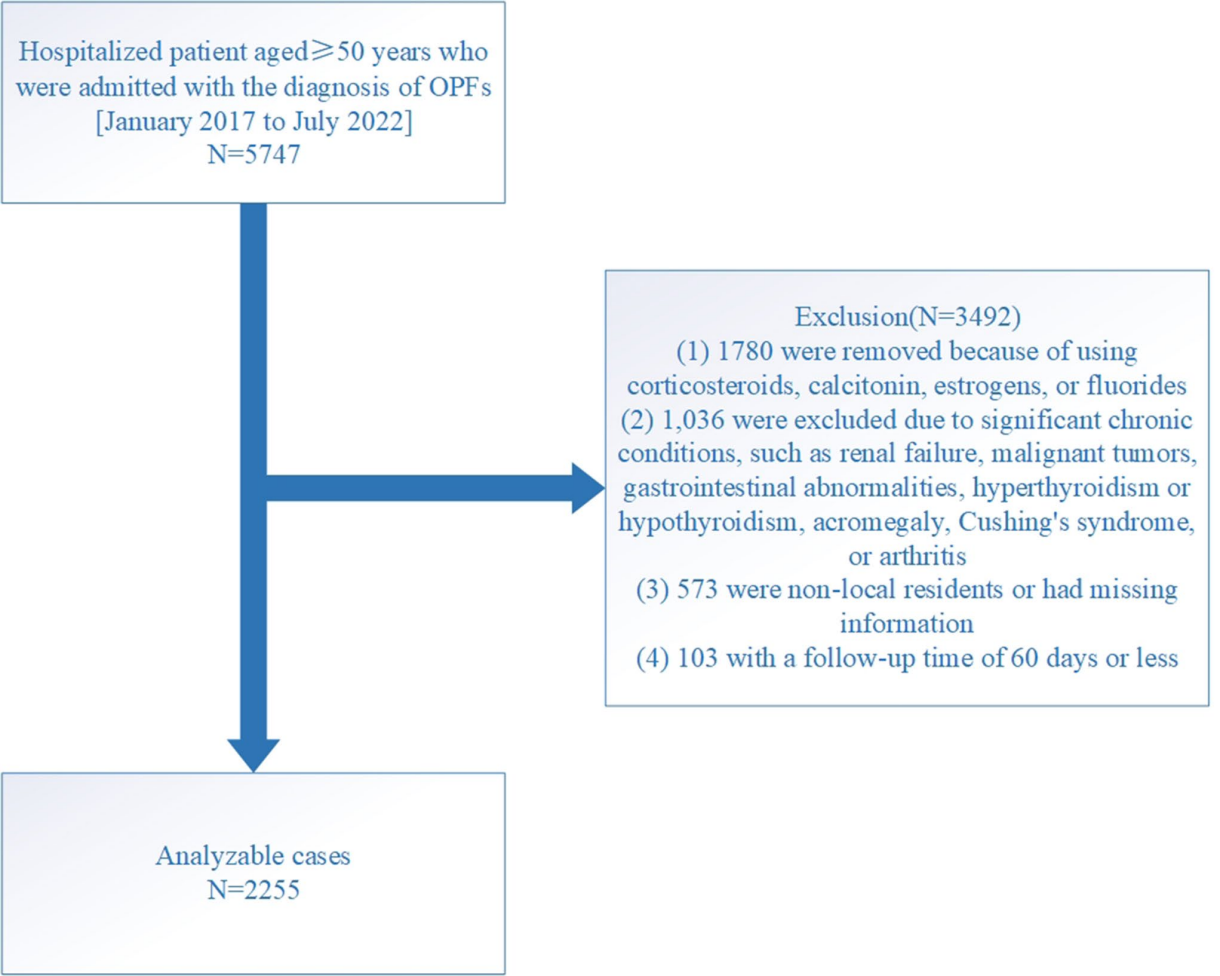
Study flow chart

This retrospective real-world study was conducted at the Affiliated Kunshan Hospital of Jiangsu University (AKHJU) and used an open enrollment design. This study utilized a database collected in advance from the Osteoporotic Fracture Registry System (OPFRS) of Jiangsu University’s Affiliated Kunshan Hospital (AKHJU). From January 1, 2017, to July 27, 2022, electronic patient data was collected from all participants aged 50 or above who were newly admitted with a diagnosis of OPFs. To refine our analysis to encompass adults with confirmed type 2 diabetes, we excluded individuals diagnosed prior to the age of 50 and those who commenced insulin therapy within one year of their diagnosis. Additionally, these patients had not sustained any fractures in the previous five years, indicating that these were their first OPFs. The enrollment process remained open throughout the entire study period. A total of 5747 successive patients with OPFs who underwent orthopedic surgery were included in this study. The following were the inclusion criteria: (1) must be at least 50 years old; (2) must have a fracture diagnosis from radiography or computed tomography, which includes fractures of the wrist, proximal humerus, lumbar vertebra, thoracic vertebra, femoral neck, and femoral trochanteric/subtrochanteric region; (3) must be receiving surgical treatment in a hospital; (4) hospital clinical data must be available; and (5) could be reached by phone. Deceased patients within the initial month of admission or those who were unreachable were excluded from the analysis. The following criteria were then used to eliminate 3492 patients from the analysis: (1) Patients who had received treatment with corticosteroids, calcitonin, estrogens, or fluorides within six months prior to entering the study (1780 cases); (2) patients with significant chronic conditions such as renal failure, malignant tumors, gastrointestinal abnormalities, hyperthyroidism or hypothyroidism, acromegaly, Cushing’s syndrome, or arthritis (1036 cases) [24]; (3) patients who were non-local residents or had incomplete data (573 cases); and (4) patients with a follow-up period of 60 days or less (103 cases). The final analysis included 2255 patients (Fig. 1), and the Declaration of Helsinki was adhered to precisely throughout the investigation. Approval for ethical review has been obtained from the AKHJU with the approved number 2021-06-015-K01. The investigators conducting the analysis were denied access to patient information. Before enrolling, we obtained informed consent and conducted all procedures in accordance with the approved guidelines.

### Exposure and outcome variables

Diabetes status was assessed based on patients’ self-reported history at baseline. The variable of outcome was refracture. The study participants were identified through the Regional Health Registration Platform (RHRP) of Kunshan City and the Population Death Registration System (PDRS) of Jiangsu Province, China. Connection of the RHRP and PDRS to the AKHJU Registration System using patient identifiers, hospital records, and relevant dates, allowed the collection of comprehensive follow-up data. Initial fractures were defined as fractures of the wrist, proximal humerus, hip, or intravertebral area, diagnosed using the International Statistical Classification of Diseases and Related Health Problems (ICD-10 nomenclature, 10th edition) with codes starting with S22, S32, S42, S52, or S72. Refracture was defined as any subsequent osteoporotic fracture occurring after an initial fracture, regardless of anatomical site. Data on refractures, including admission time, diagnosis, and relevant clinical information, were obtained from the RHRP of Kunshan City. The initial osteoporotic fracture-related hospital admission date served as the starting point for each patient. The end point was refracture. The follow-up duration was determined by analyzing the occurrence of refracture as the primary outcome event. It was defined as the time interval between the date of discharge after the initial fracture and the date of the second fracture, the patient’s transfer out of the area, or the end date of the study (July 27, 2022).

### Covariate analyses

Trained clinical researchers gathered baseline patient data. Body mass index (BMI), sex, age, smoking, drinking, the American Society of Anesthesiologists (ASA) score, hypertension, phosphorus, magnesium, sodium, hemoglobin, lymphocyte, monocyte, neutrophil, and platelet counts, albumin, calcium, aspartate aminotransferase (AST), alanine transaminase (ALT), serum uric acid (SUA), blood urea nitrogen (BUN), creatinine (Cr), total cholesterol (TC), triglyceride (TG), high-density lipoprotein (HDL), low-density lipoprotein (LDL), and fracture classification were the candidate covariates used in the analysis. The formula for calculating BMI was weight (kg) divided by height (m) squared. Anyone who has smoked during the last 12 months, whether current or past, was defined as smoking. Drinking was defined as consuming alcohol at least once per week for the previous 12 months [25]. The ASA score was utilized to assess the patients’ physical status [26]. The chief anesthesiologist of the case calculated and documented the ASA scores [27]. Blood samples were collected from the participants after an overnight fast of at least 8 h. The hematological analyzer utilized to assess the blood samples was the Sysmex XN-10 (B4). The Beckman Coulter AU5800 automated biochemical analyzer was utilized for the purpose of biochemical testing detection. The index fracture site, which comprises fractures of the wrist, proximal humerus, lumbar vertebra, thoracic vertebra, femoral neck, and femoral trochanteric/subtrochanteric region, is referred to as the fracture classification.

### Statistical analyses

All analyses were conducted using EmpowerStats (http://www.empowerstats.com) and R packages (http://www.R-project.org), with a significance limit of a two-sided *P*-value ≤ 0.05. The frequency (%) is used for categorical data. The examination of categorical data involved the use of either Pearson’s chi-square test or Fisher’s exact test for univariate analysis. The independent samples t-test was used for continuous data that followed a normal distribution, whereas the Mann-Whitney U test was applied for data that did not follow a normal distribution. Furthermore, univariate analyses were performed to investigate the relationships between refracture-related patient features and OPFs. To assess the independent association between diabetes and refracture status in patients with OPFs, Cox proportional hazard regression models were used, taking into account the effects of covariance. We presented the results from three different analyses: the unadjusted analysis, the minimum adjusted analysis, and the completely adjusted analysis, following the recommendation of the Strengthening the Reporting of Observational Studies in Epidemiology (STROBE) statement. A collinearity diagnosis was first performed using the Variance Inflation Factor (VIF). Covariate adjustments were determined based on two criteria: Criterion 1 required the inclusion or exclusion of covariates in the model, which initially included only diabetes status and refracture without covariates. The full model incorporated all potential covariates, including age, sex, BMI, smoking, drinking, magnesium, sodium, phosphorus, platelet count, hemoglobin, albumin, calcium, neutrophil count, lymphocyte count, monocyte count, ALT, AST, Cr, BUN, SUA, TC, TG, HDL, LDL, hypertension, ASA classification, and fracture classification. A covariate was considered necessary for adjustment if its inclusion or exclusion resulted in a change of ≥ 10% in the odds ratio (OR). Criterion 2 required that a covariate either met Criterion 1 or had a *P*-value < 0.1 in the univariate model. Finally, the following three models were determined: Model 2 (minimally adjusted model) was adjusted for age and BMI; Model 3 (completely adjusted model) was adjusted to account for fracture classification, platelet count, and hypertension in addition to age and BMI. Model 1 was not adjusted. We conducted a survival analysis by employing the log-rank test and examining 1-, 3-, and 5-year Kaplan-Meier (K-M) curves to determine the potential association between the existence of diabetes and the occurrence of refractures. We performed multiple subgroup analyses to evaluate the stability and potential variability of the association between diabetes and refracture risk across various covariates. Continuous variables including age, BMI, and platelet count, were divided into tertiles based on their distribution within the study population to ensure balanced sample sizes. The likelihood ratio test (LRT) was employed to analyze subgroup interactions and modifications. The *P*-value for interaction was calculated to assess whether the association between diabetes and refracture risk significantly differed across subgroup levels, with a *P*-value < 0.05 indicating a significant interaction effect.

## Results

### Patient characteristics

Table 1 displays the characteristics of individuals categorized based on their diabetes status. The study registered 2255 persons in total; 107 were in the group of patients with diabetes, 2148 in the group without diabetes. The patients’ mean age was 72.01 having a standard deviation of 10.53. The mean age of the two groups was somewhat greater in the diabetic patients group (73.11 years) than in the non-diabetic patients group (71.96 years) (standardized difference = 0.12, 95% confidence interval [CI]: -0.08 to 0.31, *P*-value = 0.269). The measurement of BMI yielded an overall mean BMI of 22.72 kg/m^2^ with a standard deviation of 3.33. The mean BMI of the diabetic patient group was 23.24 kg/m^2^, which was marginally higher than the non-diabetic patient group’s 22.70 kg/m^2^ (standardized difference = 0.17, 95% CI: -0.03 to 0.36, *P*-value = 0.098). Other measured variables, including magnesium, sodium, phosphorus, BUN, SUA, TC, TG, HDL and LDL, neutrophil, lymphocyte, monocyte, and platelet counts, hemoglobin, albumin, calcium, ALT, AST, and Cr, did not significantly differ between the two groups.

**Table 1:** Patient characteristics based on different diabetes status groups

Variables	Total	Mean ± SD^a^ / *N* (%)^b^		Standardize Difference^c^	*P*-value	*P*-value*
		Non-diabetic patients	Diabetic patients			
N	2255	2148	107			
Age, years	72.01 ± 10.53	71.96 ± 10.61	73.11 ± 8.71	0.12 (-0.08, 0.31)	0.269	0.234
BMI, kg/m^2^	22.72 ± 3.33	22.70 ± 3.34	23.24 ± 3.12	0.17 (-0.03, 0.36)	0.098	0.046
Magnesium, mmol/L	0.89 ± 0.10	0.89 ± 0.10	0.89 ± 0.09	0.01 (-0.18, 0.21)	0.903	0.990
Sodium, mmol/L	140.75 ± 2.92	140.73 ± 2.94	140.97 ± 2.68	0.08 (-0.11, 0.28)	0.412	0.238
Phosphorus, mmol/L	1.07 ± 0.22	1.07 ± 0.22	1.04 ± 0.20	0.16 (-0.04, 0.36)	0.127	0.194
Platelet count, ×10^9^/L	176.47 ± 61.61	176.58 ± 61.60	174.42 ± 62.07	0.03 (-0.16, 0.23)	0.724	0.520
Hemoglobin, g/L	125.67 ± 18.33	125.59 ± 18.45	127.33 ± 15.85	0.10 (-0.09, 0.30)	0.340	0.545
Albumin, g/L	39.98 ± 4.24	39.99 ± 4.24	39.71 ± 4.35	0.07 (-0.13, 0.26)	0.508	0.567
Calcium, mmol/L	2.21 ± 0.13	2.21 ± 0.13	2.20 ± 0.11	0.07 (-0.12, 0.27)	0.504	0.484
Neutrophil count, ×10^9^/L	6.56 ± 3.13	6.54 ± 3.11	6.85 ± 3.50	0.09 (-0.10, 0.29)	0.316	0.656
Lymphocyte count, ×10^9^/L	1.24 ± 0.54	1.24 ± 0.54	1.25 ± 0.48	0.02 (-0.18, 0.21)	0.861	0.611
Monocyte count, ×10^9^/L	0.51 ± 0.26	0.51 ± 0.26	0.49 ± 0.22	0.08 (-0.11, 0.28)	0.423	0.627
ALT, U/L	23.49 ± 20.92	23.39 ± 20.10	25.32 ± 33.48	0.07 (-0.13, 0.26)	0.355	0.635
AST, U/L	26.48 ± 24.48	26.37 ± 23.88	28.71 ± 34.47	0.08 (-0.12, 0.27)	0.338	0.414
Cr, µmol/L	65.92 ± 29.72	66.10 ± 30.21	62.34 ± 16.91	0.15 (-0.04, 0.35)	0.204	0.338
BUN, mmol/L	6.03 ± 2.43	6.03 ± 2.44	5.86 ± 2.16	0.08 (-0.12, 0.27)	0.464	0.428
SUA, µmol/L	283.23 ± 91.66	283.91 ± 91.80	269.56 ± 88.11	0.16 (-0.04, 0.35)	0.116	0.093
TC, mmol/L	4.26 ± 0.93	4.26 ± 0.94	4.30 ± 0.76	0.05 (-0.20, 0.30)	0.741	0.445
TG, mmol/L	1.24 ± 0.98	1.24 ± 0.98	1.29 ± 1.05	0.05 (-0.20, 0.30)	0.680	0.887
HDL, mmol/L	1.36 ± 0.31	1.36 ± 0.31	1.37 ± 0.28	0.04 (-0.21, 0.29)	0.752	0.688
LDL, mmol/L	2.55 ± 0.76	2.55 ± 0.77	2.54 ± 0.64	0.01 (-0.25, 0.26)	0.970	0.943
Sex				0.06 (-0.14, 0.25)	0.582	-
Female	1612 (71.49%)	1533 (71.37%)	79 (73.83%)			
Male	643 (28.51%)	615 (28.63%)	28 (26.17%)			
Smoking				0.03 (-0.16, 0.22)	0.756	-
No	2123 (94.15%)	2023 (94.18%)	100 (93.46%)			
Yes	132 (5.85%)	125 (5.82%)	7 (6.54%)			
Drinking				0.07 (-0.12, 0.27)	0.506	-
No	2189 (97.07%)	2084 (97.02%)	105 (98.13%)			
Yes	66 (2.93%)	64 (2.98%)	2 (1.87%)			
Fracture classification				0.58 (0.38, 0.77)	< 0.001	-
Thoracic vertebra	360 (15.96%)	350 (16.29%)	10 (9.35%)			
Lumbar vertebra	631 (27.98%)	605 (28.17%)	26 (24.30%)			
Wrist	101 (4.48%)	97 (4.52%)	4 (3.74%)			
Proximal humerus	260 (11.53%)	258 (12.01%)	2 (1.87%)			
Femoral neck	578 (25.63%)	543 (25.28%)	35 (32.71%)			
Femoral trochanteric/subtrochanteric	325 (14.41%)	295 (13.73%)	30 (28.04%)			
ASA recoded^d^				0.32 (0.12, 0.51)	0.014	-
1	155 (6.87%)	153 (7.12%)	2 (1.87%)			
2	1462 (64.83%)	1398 (65.08%)	64 (59.81%)			
≥ 3	638 (28.29%)	597 (27.79%)	41 (38.32%)			
Hypertension				1.39 (1.19, 1.59)	< 0.001	-
No	1849 (82.00%)	1819 (84.68%)	30 (28.04%)			
Yes	406 (18.00%)	329 (15.32%)	77 (71.96%)			

^a^Continuous variables

^b^Categorical variables

^c^Standardized differences of < 0.10 for a given covariate indicate a relatively small imbalance

^d^Higher scores indicate more severe physical status

*P*-value*: Kruskal−Wallis Rank Test for continuous variables, Fisher Exact for categorical variables with Expects < 10

Abbreviations: SD, standard deviation; BMI, body mass index; ALT, alanine aminotransferase; AST, aspartate aminotransferase; Cr, creatinine; BUN, blood urea nitrogen; SUA, serum uric acid; TC, total cholesterol; TG, triglycerides; HDL, High-density lipoprotein; LDL, Low-density lipoprotein; ASA, American Society of Anesthesiologists

Sex did not significantly differ across the groups in terms of categorical factors (standardized difference = 0.06, 95% CI: -0.14 to 0.25, *P*-value = 0.582). The female gender constituted the majority of patients in both the non-diabetic patient group (71.49%) and the diabetes patient group (73.83%). Similarly, there were no significant differences in drinking or smoking between the two groups. The groups differed significantly in terms of fracture classification, which was classified as thoracic vertebra, lumbar vertebra, wrist, proximal humerus, femoral neck, and femoral trochanteric/subtrochanteric (standardized difference = 0.58, 95% CI: 0.38 to 0.77, *P*-value < 0.001). A significant difference between the groups was also evident in the ASA score (standardized difference = 0.32, 95% CI: 0.12 to 0.51; *P*-value = 0.014). There was a significant difference in the occurrence of hypertension across the groups, with a higher rate in the group of individuals with diabetes (standardized difference = 1.39, 95% CI: 1.19 to 1.59, *P*-value < 0.001).

### Univariate analysis of factors associated with refracture status

The results of the univariate analysis examining variables associated with refracture status are presented in Table S1. Significantly, the analysis highlights that diabetes mellitus is associated with an increased risk of refracture [hazard ratio (HR) = 2.02, 95% CI: 1.05 to 3.86, *P*-value = 0.034]. Other factors such as age and platelet count also showed statistically significant associations with refracture risk, while variables like BMI, magnesium, and lipid components did not show significant correlations. The table also includes categorical assessments of sex, smoking, drinking, and fracture classification, with specific HRs indicating the relative risk of refracture among these different categories.

### Evaluation of the relationship between diabetes and refracture status

**Fig. 2:**
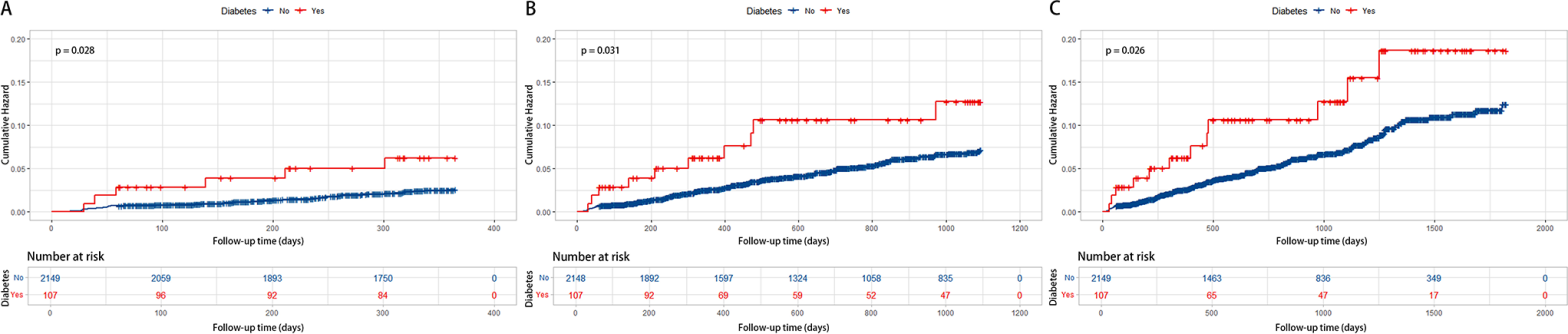
Cumulative hazard of refracture for OPFs patients with diabetes (red line) compared with OPFs patients without diabetes (blue line) at 1 year (A), 3 years (B), and 5 years (C) follow-up. Adjusted covariates included age, sex, BMI, hypertension, platelet count, and fracture classification. OPFs, osteoporotic fracture; BMI, body mass index

The examination of the association between diabetes and refracture status during three different follow-up periods—1, 3, and 5 years—is shown in Table 2. The table includes three models: the non-adjusted model, the minimally adjusted model, and the fully adjusted model. Diabetes was consistently associated with a considerably elevated risk of refracture across all models and throughout all follow-up periods. In the fully adjusted model, this link remained significant even after controlling for possible confounders including age, sex, BMI, hypertension, platelet count, and fracture classification. For the follow-up periods of 1, 3, and 5 years, the HRs in the fully adjusted model are 2.83 (95% CI: 1.09 to 7.39, *P*-value = 0.033), 2.65 (95% CI: 1.27 to 5.52, *P*-value = 0.009), and 2.72 (95% CI: 1.39 to 5.32, *P*-value = 0.004), in that order. These findings indicate that individuals with diabetes are at a significantly higher risk of experiencing refracture, with the risk being more than double compared to those without diabetes. The relationship between diabetes status and cumulative refracture rates was evaluated using Kaplan–Meier curves (Fig. 2). There is a significant difference in the rates of refracture between the groups with and without diabetes. The diabetic group has a significantly elevated risk of refracture in comparison to the non-diabetic group. The Kaplan-Meier curves for the 1-year, 3-year, and 5-year follow-up periods display the same trend.

**Table 2:** Association between diabetes and refracture status in different models

Follow-up period	Diabetes	No. of Events	Refracture Rate (%)	HR (95% CI) *P*-value		
				Non-Adjusted Model	Minimally-Adjusted Model	Fully-Adjusted Model
1 year	No	50	2.3	Reference	Reference	Reference
	Yes	6	5.6	2.50 (1.07, 5.84) 0.0336	2.50 (1.07, 5.86) 0.0344	2.83 (1.09, 7.39) 0.0332
3 years	No	104	4.8	Reference	Reference	Reference
	Yes	10	9.3	2.02 (1.05, 3.86) 0.0342	1.92 (1.00, 3.68) 0.0492	2.65 (1.27, 5.52) 0.0091
5 years	No	128	6.0	Reference	Reference	Reference
	Yes	12	11.2	1.94 (1.07, 3.50) 0.0285	1.86 (1.03, 3.36) 0.0403	2.72 (1.39, 5.32) 0.0035

Outcome variable: Refracture status, Exposed variables: Diabetes status

Minimally adjusted model adjusted for age, sex, and BMI

Fully adjusted model adjusted for age, sex, BMI, hypertension, platelet count, and fracture classification

Abbreviations: HR, Hazard Ratio; BMI, body mass index

### Subgroup analysis

This study stratified all subgroups by age, sex, BMI, fracture classification, hypertension and platelet count to further verify the reliability of the outcome results in the fully adjusted model when potential confounding variables were represented. All analyses, except for the subgroup variable, were adjusted for these covariates. Table 3 demonstrates an extremely uniform pattern. The age group 77–105 showed a trend toward higher risk in the age group subgroup analysis (HR = 3.67, 95% CI: 1.14 to 11.78, *P*-value = 0.029). Within the subgroup analysis based on sex, diabetes and refracture were found to be significantly associated in females (HR = 2.79, 95% CI: 1.28 to 6.06, *P*-value = 0.010), but not in men (HR = 1.87, 95% CI: 0.20 to 17.07, *P*-value = 0.579). The study revealed a strong association between diabetes and refracture in the subgroup analysis of individuals with a BMI between 21.24 and 24.15 (HR = 4.83, 95% CI: 1.63 to 14.33, *P*-value = 0.005). However, this correlation was not observed in the other BMI groups. No significant correlations were observed between diabetes and refracture in subgroup analyses that considered fracture classification, hypertension status, and platelet count.

**Table 3:** Subgroup analyses exploring the association between diabetes and refracture status

Subgroup	*N*	No. of Events	Refracture Rate (%)	HR (95% CI) *P*-value	*P*-value for interaction
AGE group					
50–66	715	26	3.64	1.21 (0.15, 9.94) 0.8608	0.6213
67–76	743	38	5.11	2.94 (0.96, 9.00) 0.0597	
77–105	797	50	6.27	3.67 (1.14, 11.78) 0.0289	
Sex					
Female	1612	95	5.89	2.79 (1.28, 6.06) 0.0098	0.7406
Male	643	19	2.95	1.87 (0.20, 17.07) 0.5794	
BMI group					
13.52–21.23	752	38	5.05	1.16 (0.14, 9.66) 0.8889	0.3393
21.24–24.15	751	39	5.19	4.83 (1.63, 14.33) 0.0046	
24.16–40	752	37	4.92	1.79 (0.57, 5.65) 0.3193	
Fracture classification					
Thoracic vertebra	360	23	6.39	2.50 (0.30, 20.87) 0.3986	0.7818
Lumbar vertebra	631	36	5.71	3.77 (0.94, 15.12) 0.0611	
Wrist	101	3	2.97	0.00 (0.00, Inf) 0.9995	
Proximal humerus	260	10	3.85	0.00 (0.00, Inf) 0.9997	
Femoral neck	578	28	4.84	3.01 (1.00, 9.10) 0.0505	
Femoral trochanteric/subtrochanteric	325	14	4.31	1.13 (0.13, 9.52) 0.9099	
Hypertension group					
No	1849	94	5.08	3.53 (1.27, 9.78) 0.0154	0.4966
Yes	406	20	4.93	2.12 (0.78, 5.78) 0.1408	
Platelet group					
10–146	731	30	4.1	3.52 (0.87, 14.31) 0.0787	0.7726
147–194.6	736	42	5.71	1.87 (0.61, 5.75) 0.2776	
195–751	745	38	5.1	2.55 (0.69, 9.47) 0.1608	

Adjusted for age, sex, BMI, fracture classification, hypertension, and platelet count, except the subgroup variable

Abbreviations: HR, hazard ratio; BMI, body mass index

## Discussion

This 5-year retrospective cohort study examined the occurrence of refracture in 2255 hospitalized patients with OPFs between a group of patients with diabetes and a group without diabetes. Several subgroup analyses were conducted to assess the relationship between patient characteristics, refracture, and diabetes status. The findings indicated a significant and evident direct relationship between diabetes status and refracture in patients with OPFs, considering the inclusion of additional variables in the fully adjusted model. According to the findings, those with diabetes are over twice as likely to experience a refracture than people without the disease.

Despite some data indicating that diabetes decreases bone density [28–30], the risk of fracture and its clinical significance in individuals with diabetes remain controversial [31]. Diabetes and the risk of fracture have been established correlated by numerous major prospective studies and meta-analyses [32–36]. However, the information regarding the incidence of fractures in individuals with diabetes is still conflicting in a small number of other research; some findings suggest a lower risk or no association [37, 38]. Patients with diabetes who had recently received their diagnosis had a lower fracture risk in a subgroup analysis of a large Rotterdam research. However, it was discovered that the risk rose in patients with late-stage diabetes who were getting treatment [39]. Interestingly, a prospective cohort study conducted in Spain found that among 202 overweight white women with type 2 diabetes over 65, the risk of fractures did not increase [40]. Additionally, a different Swedish study found that older diabetic women who did not have severe renal impairment also had lower bone turnover and higher bone mass. Low bone turnover and high bone mass, however, are unlikely to have a major effect on fracture susceptibility [41]. The reported differences in outcomes from Western studies are believed to be influenced by variations in race and geographic region, while the precise causes remain unidentified. The study specifically examined Asian individuals with OPFs and discovered a significant and independent association between the presence of diabetes and the probability of experiencing refracture.

While numerous studies have focused on the initial fracture risk in diabetes, research specifically examining the refracture risk in diabetic patients with previous osteoporotic fractures is relatively limited. Our findings demonstrated that diabetes significantly increases the risk of subsequent fractures in patients who have already experienced an initial osteoporotic fracture. This higher refracture risk in diabetic patients may be attributed to several mechanisms that particularly affect bone healing and recovery after the initial fracture.

The mechanism behind the increased risk of refracture in patients with diabetes differs from that of typical osteoporosis and may be particularly relevant after an initial fracture. Patients with diabetes face unique challenges in bone healing and recovery due to several factors. These include decreased cortical density, altered bone geometry, faster bone loss, microdamage accumulation at low bone turnover regions, and AGEs accumulation [42]. Specifically, people with diabetes frequently lose bone at a faster pace than people without the disease [43]. This is coupled with a decline in cortical density, which further reduces the bone strength [44]. Changes in the structure of the bone may potentially increase the risk of fracture because they can affect the way stresses are distributed throughout the bone [45]. Furthermore, in areas with low bone turnover, the accumulation of microdamage can erode the structural integrity of the bone, raising the risk of fractures [41]. The accumulation of AGEs is another crucial element. AGEs, which are proteins or lipids that have undergone glycation as a result of being exposed to sugars, may significantly change the properties of collagen. Tensile strength in bone is provided by the essential protein collagen. The bone becomes more brittle as AGEs change its characteristics. The paradoxical rise in fracture risk that is seen in diabetics is further exacerbated by this process [46]. An increased chance of falls or other nonskeletal risk factors associated with diabetes may be another mechanism causing an increased risk [47]. Hypoglycemia, a side effect of some diabetic medications, can lead to disorientation and dizziness, which raises the risk of falls [48]. All these factors contribute significantly to the higher risk of subsequent fractures that diabetics experience. Our findings along with those of others, demonstrate that factors other than BMD affect subsequent fracture risk, particularly in individuals with diabetes.

Additionally, due to the extended duration of our follow-up period, our study included individuals with long-standing prevalent diabetes. Because of this, our study participants were more likely to have diabetes complications and to be using antidiabetic drugs, which may have had an impact on the observed incidence of bone fractures. Individuals with diabetes have an increased risk of developing cardiovascular, renal, and neuropathy disorders [49]. Diabetes-related complications may impair the healing process following an initial fracture, thereby increasing the risk of subsequent fractures [50, 51]. Furthermore, the healing process in diabetic patients may be further hindered by the effects of medication. Although insulin obviously has an anabolic action in bone, which helps some of the beneficial effects on BMD, the skeletal benefits of insulin treatment are still controversial [52]. Furthermore, thiazolidinediones (TZDs) such as pioglitazone and rosiglitazone, are among the various groups of oral antidiabetic medications that still have the risk of adversely affecting bone health. TZDs function as agonists for the nuclear receptor known as peroxisome proliferator-activated receptor gamma (PPARγ), which is necessary for adipogenesis and insulin sensitivity. While PPARγ activation can improve insulin resistance, it also promotes the differentiation of mesenchymal stem cells into adipocytes rather than osteoblasts. This alteration in cellular specialization reduces the production of new bone and bone cells [53, 54]. This could be particularly detrimental for patients recovering from an initial fracture, and may contribute to an increased risk of refracture.

The development of secondary prevention measures is essential because individuals with diabetes have a risk of refracture that is more than double that of those without the disease. From this point, the clinical implications of our findings emphasize the need for enhanced secondary fracture prevention strategies in patients with diabetes who have experienced an initial osteoporotic fracture. These patients require particularly careful monitoring and management to prevent subsequent fractures. Regular monitoring of bone health and periodic evaluations of BMD and other indicators of bone turnover should form part of patients with diabetes’s normal treatment. Additionally, very important for secondary prevention efforts are lifestyle changes that improve general bone health. These may include the promotion of weight-bearing exercises, ensuring an adequate intake of calcium and vitamin D, maintaining a healthy weight, and advocating smoking cessation and moderate alcohol consumption. Examining each patient’s medication plan in relation to their individual fracture risk profile is also very important. If a patient has a high risk of fractures or has already had one, healthcare professionals may think about changing their diabetes prescription to those less likely to affect bone health or include osteoporosis drugs into their treatment plan. Understanding the increased fracture risk in people with diabetes and using proactive steps to prevent it allows us to apply comprehensive multifactorial approaches tailored depending on patient profiles. This strategic approach might considerably raise patient outcomes.

This study has several limitations. Firstly, due to the retrospective nature of our study, we were unable to gather data on key factors such as the length of diabetes, occurrences of hypoglycemia, instances of falls, specific types of antidiabetic medications, comorbidities, and complications related to diabetes. These variables may be essential for comprehending the correlation between diabetes and the risk of refracture. Additionally, our study solely focused on Chinese individuals, which restricts the applicability of our findings to other demographics and ethnicities. Furthermore, a notable limitation of our study is the relatively small number of diabetic patients (*n* = 107) compared to non-diabetic patients (*n* = 2148), which reduces the statistical power and highlights the need for future studies with larger, multicenter cohorts to enhance the model. Additionally, since the study period (January 2017 to July 2022) slightly exceeded five years, only a subset of participants had the potential for a full 5-year follow-up, potentially affecting the robustness of our long-term findings. Future study should focus on collecting extensive data in a systematic manner, incorporating a wider range of patients, and performing analyses that take into account the presence of various comorbid conditions. This will lead to a more detailed and nuanced understanding of the connection between diabetes and the risk of fractures.

Nevertheless, our study contained some significant advantages. The study sample comprised Chinese individuals aged 50 years or older residing in metropolitan areas. The study was conducted as a retrospective analysis of real-world data, with follow-up assessments at 1, 3, and 5 years. Regarding the study results, three independent models were employed to completely examine the relationship between diabetes and refracture status. These models were adjusted for several potential confounding variables such as age, sex, BMI, hypertension, platelet count, and fracture classification. Additionally, we conducted subgroup analyses and found that the results were similar across several models and subgroups, indicating the stability and reliability of our findings.

## Conclusions

This study offers valuable insights into the correlation between diabetes and the risk of refracture in individuals with OPFs. The results demonstrate a significant and positive correlation between the risk of refracture and diabetes, suggesting that individuals with diabetes are more than twice as likely to experience a refracture compared to those without diabetes. The results indicate the need for comprehensive preventive measures, including lifestyle changes that enhance overall bone health, a thorough evaluation of medication schedules based on each patient’s specific fracture risk profile, and potentially the inclusion of osteoporosis medications in the treatment plans of individuals at high risk. Additional study is necessary to validate these findings in different populations and to investigate the underlying mechanisms more thoroughly.

## Electronic supplementary material

Below is the link to the electronic supplementary material.

Supplementary Material 1

Supplementary Material 2

## Data Availability

Data is provided within the supplementary information files.
