# Supplementary material for: Correlation between diabetes mellitus and refracture risk in patients with osteoporotic fractures: a retrospective cohort study
Source: Aging Clin Exp Res. 2025 Mar 13;37(1):85. doi: 10.1007/s40520-024-02917-1 (PMC11903520; doi:10.1007/s40520-024-02917-1)
Supplement: Supplementary file 1 — Supplementary Material 1 [file 40520_2024_2917_MOESM1_ESM.docx]

**Table S1** Univariate analyses of factors associated with refracture status

| **Variables** | **Statistics** | **HR (95% CI) *P*-value** |
| --- | --- | --- |
| Age, years | 72.01 ± 10.53 | 1.03 (1.01, 1.05) 0.001 |
| BMI, kg/m^2^ | 22.72 ± 3.33 | 0.98 (0.93, 1.04) 0.528 |
| Magnesium, mmol/L | 0.89 ± 0.10 | 0.21 (0.03, 1.41) 0.107 |
| Sodium, mmol/L | 140.75 ± 2.92 | 0.97 (0.91, 1.03) 0.278 |
| Phosphorus, mmol/L | 1.07 ± 0.22 | 1.40 (0.61, 3.23) 0.426 |
| Platelet count, ×10^9^/L | 176.47 ± 61.61 | 1.00 (1.00, 1.01) 0.032 |
| Hemoglobin, g/L | 125.67 ± 18.33 | 0.99 (0.98, 1.00) 0.269 |
| Albumin, g/L | 39.98 ± 4.24 | 1.00 (0.96, 1.04) 0.950 |
| Calcium, mmol/L | 2.21 ± 0.13 | 0.70 (0.17, 2.93) 0.621 |
| Neutrophil count, ×10^9^/L | 6.56 ± 3.13 | 1.02 (0.96, 1.08) 0.447 |
| Lymphocyte count, ×10^9^/L | 1.24 ± 0.54 | 1.08 (0.77, 1.51) 0.659 |
| Monocyte count, ×10^9^/L | 0.51 ± 0.26 | 1.11 (0.54, 2.27) 0.780 |
| ALT, U/L | 23.49 ± 20.92 | 1.00 (0.99, 1.01) 0.905 |
| AST, U/L | 26.48 ± 24.48 | 1.00 (0.99, 1.01) 0.772 |
| Cr, μmol/L | 65.92 ± 29.72 | 1.00 (0.99, 1.01) 0.850 |
| BUN, mmol/L | 6.03 ± 2.43 | 0.97 (0.88, 1.06) 0.467 |
| SUA, μmol/L | 283.23 ± 91.66 | 1.00 (1.00, 1.00) 0.654 |
| TC, mmol/L | 4.26 ± 0.93 | 0.89 (0.66, 1.21) 0.466 |
| TG, mmol/L | 1.24 ± 0.98 | 0.91 (0.65, 1.26) 0.568 |
| HDL, mmol/L | 1.36 ± 0.31 | 0.73 (0.29, 1.85) 0.511 |
| LDL, mmol/L | 2.55 ± 0.76 | 0.93 (0.64, 1.34) 0.685 |
| Sex |  |  |
| Female | 1612 (71.49%) | Reference |
| Male | 643 (28.51%) | 0.52 (0.32, 0.85) 0.009 |
| Smoking |  |  |
| No | 2123 (94.15%) | Reference |
| Yes | 132 (5.85%) | 0.00 (0.00, Inf) 0.993 |
| Drinking |  |  |
| No | 2189 (97.07%) | Reference |
| Yes | 66 (2.93%) | 0.00 (0.00, Inf) 0.992 |
| Fracture classification |  |  |
| Thoracic vertebra | 360 (15.96%) | Reference |
| Lumbar vertebra | 631 (27.98%) | 0.89 (0.53, 1.50) 0.650 |
| Wrist | 101 (4.48%) | 0.48 (0.14, 1.61) 0.234 |
| Proximal humerus | 260 (11.53%) | 0.55 (0.26, 1.15) 0.112 |
| Femoral neck | 578 (25.63%) | 0.84 (0.49, 1.46) 0.545 |
| Femoral trochanteric/subtrochanteric | 325 (14.41%) | 0.72 (0.37, 1.41) 0.339 |
| ASA recoded |  |  |
| 1 | 155 (6.87%) | Reference |
| 2 | 1462 (64.83%) | 1.13 (0.52, 2.46) 0.749 |
| ≥3 | 638 (28.29%) | 1.30 (0.57, 2.94) 0.530 |
| Hypertension |  |  |
| No | 1849 (82.00%) | Reference |
| Yes | 406 (18.00%) | 1.02 (0.63, 1.65) 0.933 |
| Diabetes |  |  |
| No | 2148 (95.25%) | Reference |
| Yes | 107 (4.75%) | 2.02 (1.05, 3.86) 0.034 |

Abbreviations: SD, standard deviation; BMI, body mass index; ALT, alanine aminotransferase; AST, aspartate aminotransferase; Cr, creatinine; BUN, blood urea nitrogen; SUA, serum uric acid; TC, total cholesterol; TG, triglycerides; HDL, High-density lipoprotein; LDL, Low-density lipoprotein; ASA, American Society of Anesthesiologists.
